# Genome editing of oncogenes with ZFNs and TALENs: caveats in nuclease design

**DOI:** 10.1186/s12935-018-0666-0

**Published:** 2018-10-22

**Authors:** Sumitra Shankar, Ahalya Sreekumar, Deepti Prasad, Ani V. Das, M. Radhakrishna Pillai

**Affiliations:** Rajiv Gandhi Centre for Biotechnology, Thiruvananthapuram, India

**Keywords:** Zinc-finger nucleases, TALEN, HPV, Cervical cancer, Gene editing

## Abstract

**Background:**

Gene knockout technologies involving programmable nucleases have been used to create knockouts in several applications. Gene editing using Zinc-finger nucleases (ZFNs), Transcription activator like effectors (TALEs) and CRISPR/Cas systems has been used to create changes in the genome in order to make it non-functional. In the present study, we have looked into the possibility of using six fingered CompoZr ZFN pair to target the E6 gene of HPV 16 genome.

**Methods:**

HPV 16^+ve^ cell lines; SiHa and CaSki were used for experiments. CompoZr ZFNs targeting E6 gene were designed and constructed by Sigma-Aldrich. TALENs targeting E6 and E7 genes were made using TALEN assembly kit. Gene editing was monitored by T7E1 mismatch nuclease and Nuclease resistance assays. Levels of E6 and E7 were further analyzed by RT-PCR, western blot as well as immunoflourescence analyses. To check if there is any interference due to methylation, cell lines were treated with sodium butyrate, and Nocodazole.

**Results:**

Although ZFN editing activity in yeast based MEL-I assay was high, it yielded very low activity in tumor cell lines; only 6% editing in CaSki and negligible activity in SiHa cell lines. Though editing efficiency was better in CaSki, no significant reduction in E6 protein levels was observed in immunocytochemical analysis. Further, in silico analysis of DNA binding prediction revealed that some of the ZFN modules bound to sequence that did not match the target sequence. Hence, alternate ZFN pairs for E6 and E7 were not synthesized since no further active sites could be identified by in silico analyses. Then we designed TALENs to target E6 and E7 gene. TALENs designed to target E7 gene led to reduction of E7 levels in CaSki and SiHa cervical cancer cell lines. However, TALEN designed to target E6 gene did not yield any editing activity.

**Conclusions:**

Our study highlights that designed nucleases intended to obtain bulk effect should have a reasonable editing activity which reflects phenotypically as well. Nucleases with low editing efficiency, intended for generation of knockout cell lines nucleases could be obtained by single cell cloning. This could serve as a criterion for designing ZFNs and TALENs.

**Electronic supplementary material:**

The online version of this article (10.1186/s12935-018-0666-0) contains supplementary material, which is available to authorized users.

## Background

The field of gene therapy has advanced a lot with the advent of programming nucleases. Genome engineering involves the use of DNA-binding modules that can be combined with nucleases to impact genomic structure and function. This largely depends on the DNA-binding specificity and affinity of designed zinc-finger and TALE (Transcription activator like effectors) proteins. Recently CRISPR/Cas system has also been used for genome editing experiments [1–4]. Such a knockout technology offers advantage over other approaches such that a specific area of the DNA could be modified with a single dose of administration of these molecules, thereby making permanent change in the genome at once and making it non-functional. Recent reports have shown that programmable nucleases could be used to correct mutations in diseases such as SCID (Severe Combined Immunodeficiency), Hemophilia, and sickle cell disease, etc. [5–7]. These tools have also been used to develop null phenotypes or make a gene non-functional by Non-Homologous End Joining (NHEJ)-mediated double strand break repair which leads to the introduction of small insertions or deletions at the targeted site, resulting in knockout of gene function via frame-shift mutations. In order to make them suitable for clinical applications, DNA binding specificity and affinity plays an important role. In addition to this, the nucleases have been designed as heterodimers to enhance cleavage specificity and reduced toxicity [8, 9].

The possibility of virtually targeting any region in the genome has attracted several researchers to use genome editing tools in their field of study. Genome editing is done using sequence-specific DNA-binding domains fused to a non-specific DNA cleavage module which allow efficient and precise modifications in the gene of interest causing DNA double-strand breaks (DSBs), thereby stimulating the cellular DNA repair mechanisms [10]. Zinc-finger (ZFNs) and transcription activator-like effector proteins (TALENs) are two such DNA binding proteins that could be engineered to target specific gene of interest. Efficiency of targeted gene editing depends on the affinity and specificity of DNA-binding of these nucleases. ZFNs have been successfully applied to excise HIV-I proviral DNA [11] and this has opened up new possibilities of ZFN/TALEN application to target other viral genomes as well. There are studies which have also shown that by combining zinc finger proteins with cell penetrating peptides, HPV-18 DNA replication was inhibited. This capability of these zinc finger fusion proteins to function as potent anti-viral drugs in transient replication assays has also been demonstrated [12]. ZFNs have also been used to target Hepatitis B Virus which prevent viral reactivation and replication [13, 14]. Similarly, TALEN was used to knockout *APOB* gene in order to investigate its role in Hepatitis C Virus infection [15]. These examples show that ZFNs or TALENs could be used to achieve a knockout since they share the same mode of action.

There are several publicly available open access selection approaches created to design zinc finger proteins as well as TALENs with customized specificity [16–19]. A number of software tools have been developed in which the binding specificity of hundreds of artificial and natural zinc fingers have been characterized by several research groups. In spite of advances in nuclease designs, there is still no assurance that the pair would work effectively for that particular target region in a particular system.

Here, ZFNs and TALENs were used to target Human Papillomavirus oncogenes. CompoZr ZFNs (from Sigma) were used to target *E6* gene of HPV 16. Although gene editing activity of the six-fingered CompoZr ZFNs in yeast based MEL-I assay showed activity greater than 50%, editing was found to be low in SiHa and CaSki cervical cancer cell lines was observed by endonuclease assays and immunofluorescence experiments. We looked at DNA binding prediction which revealed that sequences bound by ZFN modules did not match the exact target site hence, we screened for suitable ZFN pairs that could edit *E6* gene using publicly available computational tools. Since no useful target sites were obtained for ZFNs, we designed TALENs targeting *E6* which also did not yield any editing activity in these cell lines. We recently showed that TALENs successfully edited *E7* gene in SiHa cells [20]. We extended this editing study to another HPV^+ve^ cell line CaSki and found similar editing efficiency. There was significant reduction in mRNA and protein levels of E7 after TALEN treatment. In the present study, we have compared the downstream effects of E7 and E6 oncogenes using these synthetic nucleases. From our study we observed that while E7 targeting by TALENs was very successful, E6 targeting by both ZFNs and TALENs turned out to be ineffective.

## Materials and methods

### Cell lines used

Two HPV 16^+ve^ cell lines; SiHa and CaSki were used for experiments. These cell lines were grown in DMEM containing 10% FBS in CO_2_ incubator at 37 °C. The presence of HPV 16 was confirmed by analyzing the presence of *E6* gene by PCR and also sequencing of *E6* gene in the genomic DNA was isolated from both the cell lines.

### Transfection

Transfection was done using Lipofectamine LTX plus reagent (Cat # 15338100, Invitrogen) as per manufacturer’s instructions. The cells were seeded and cultured to reach 60–70% confluency and then transfected with ZFN plasmids (~ 5 µg) using lipofectamine LTX reagent and plus reagent. Briefly, the lipofectamine LTX-plus-DNA complex was prepared in opti-MEM I (Life technologies, USA) and incubated at room temperature for 25 min and then added to the culture plate in a dropwise manner. Plasmid carrying GFP (pGFPmax, Amaxa) was transfected as control to monitor transfection efficiency. Genomic DNA was isolated from both the cell lines and subjected to further analyses.

### Preparation of ssODN

The ssODNs donor template sequences which have a BAMH1 site (underlined) *ATGATATAATATTAGAATGTGTGTACTGCAAGCAACAGTTACTGCGGATCCGAGGTATATGACTTTGCTTTTCGGGATTTATGCATAGTATATAGA* were synthesized as normal oligonucleotides and purified by PAGE (Sigma). ssODNs were diluted with RNase free water to 100 μM, and stored at − 20 °C. For ssODN nucleofection, 10 μM of working solution of ssODN was mixed with 5 μg of *ZFN* plasmids before nucleofection. Cells were grown at 37 °C and 5% CO_2_ after nucleofection. For nucleofection, solution R (82 μl) and supplement of 18 μl were mixed with cells and program A-028 was used (Amaxa) was used.

### TALEN assembly and sequencing

This was done using *Sidansai Biotechnology* TALEN assembly kit (Cat# GL201305-3), according to the vendor’s protocol. Briefly, TALE modules and their backbone vector for right arm and left arm were mixed with solution 1, 2 and 3 and keep the tubes in the thermal cycler with a ligation program 37/25° for 20 cycles. Then the ligation mix was transformed into DH5α strain. Colony PCR was done to confirm the positive clones. Then plasmids were isolated from positive clones and sequenced using ABI 310 mix (Bigdye terminator v 3.1, Applied Biosystems). TALEN vector backbone contained the following elements CMV-SP6-3XFlag-NLS-N′-[TALE]-C′-FokI-IRES-puro-pA. TALEN targeting *E6* gene contained 18 modules on both arm and a spacer of 19 nucleotides while TALEN targeting *E7* contained 18 modules on both arm and a spacer of 21 nucleotides.

### CompoZr zinc finger nucleases

ZFNs targeting *E6* gene were designed and constructed by Sigma-Aldrich (St Louis, MO, USA, Lot Number: 08231013MN). The selection of modules, cloning and validation of the ZFNs by Yeast MEL-I assay [21] was performed by Sigma-Aldrich. ZFN design involved using an archive of pre-validated two-finger and one-finger modules. ZFNs used for this study are heterodimers containing Fok I variant EL, KK. The target region was scanned for several positions for which suitable modules existed in the archive. pZFN plasmid contains CMV promoter and BGH polyA tail.

### T7E1 mismatch endonuclease assay

In order to monitor the gene editing, T7E1 mismatch nuclease assay was carried out [22]. For this, genomic DNA was isolated from both the cell lines and PCR for *E6* gene was performed in control and ZFN treated samples. Similarly, PCR was done in TALEN treated cells targeting E6. 10 µl of the PCR products from control and treated samples were taken and subjected to the following program in a thermocycler.

95 °C 10 min

95–85 °C − 2 °C/s

85–25 °C − 0.1 °C/s

4 °C ∞

After the reaction was complete, 1 μl of T7E1 (Cat # M0302S) enzyme and 2 µl of 10× buffer 2 was added to the PCR samples and incubated at 37 °C for 20 min. The digestion product was run in a 2.0% agarose gel and the result was recorded in the gel documentation system (Bio-Rad).

### Nuclease resistance assay

Nuclease resistance assay was also done to confirm the gene editing in SiHa and CaSki cells. Briefly, *E6* gene was amplified from DNA extracted from control and treated samples. 5 µl of the PCR product was digested with HPyChIV4 (Cat # R0619S) enzyme. The interpretation of result is that the control DNA gets completely digested whereas the treated population of cells which have undergone gene editing will give an uncut product. The intensity of the uncut product was analyzed by image J software to measure ZFN editing activity.

### RT-PCR analysis

Isolation of total RNA and cDNA synthesis were carried out as per manufacturer’s instructions. RNA was isolated from the SiHa cells using DNA/RNA kit (Qiagen Cat # 80204) and ~ 1 μg of RNA was transcribed into cDNA using MMLV reverse transcriptase (Promega Cat# M1701), 0.5 µl of dNTPs, 0.5 µl of RNase inhibitor, 4 µl of reverse random primer in a total volume of 20 μl. Specific transcripts were amplified using gene-specific primers:

*HPV E6*

Forward: 5′-ATGCATGGAGATACACCTACATTG-3′,

Reverse: 5′-CATTACATCCCGTACCCTCTTC-3′;

*HPV E7*

Forward: 5′-ATGCACCAAAAGAGAACTGCAATGT-3′,

Reverse: 5′-TTACAGCTGGGTTTCTCTACGTG-3′;

*β*-*actin*

Forward: 5′-AGACTTCGAGCAGGAGATG-3′,

Reverse: 5′-CTTGATCTTCATGGTGCTAGG-3′

Amplifications were carried out for 25 cycles on a Veriti Thermal cycler (Applied Biosystems) and the products were visualized by ethidium bromide staining after electrophoresis on 2% agarose gel. The bands corresponding to specific transcripts were scanned using a densitometer and normalized against the values corresponding to *β*-*actin* transcript bands.

### Immunofluorescence

Further to confirm whether ZFN-mediated gene editing resulted in reduction of *E6* and *E7* expression levels in vitro, an immunocytochemical analysis was carried out using specific antibodies. For this, 72 h after treatment, SiHa cells were washed with 1× PBS and fixed in 4% PFA. Cells were then permeabilized with acetone: methanol (1:1) for 20 min and blocked with 3% BSA for 1 h followed by overnight incubation anti-E6 (1:100, Santacruz, Cat # SC460) and anti-E7 (1:100, Santacruz, Cat # Sc 6981) antibody. After washing three times with 1× PBS, the cells were incubated with secondary bodies in appropriate dilutions (1:400, anti-mouse FITC; Life technologies, Cat# A21200). The cells were counterstained with DAPI (D1306, Thermofisher Scientific) and mounted in 80% glycerol and sealed. Images were taken in confocal laser scanning microscope (Nikon).

In order to further account for the editing, we assessed the expression of 53BP1, a marker for DNA damage by immunocytochemical analysis. Fourteen hours after treatment SiHa cells were washed with PBS and fixed in 4% PFA. Cells were permeabilized with acetone: methanol (1:1) for 20 min and blocked with 3% BSA for 1 h followed by overnight incubation of anti-53BP1 (1:100, Cell signaling Cat # 4937). Cells were then washed with PBS followed by 1 h incubation with anti-rabbit FITC (1:100, Sigma Cat # F9887) and were counterstained with DAPI. Cells were then washed and mounted in 80% glycerol. Images were taken in confocal laser scanning microscope (Nikon).

### Statistical analysis

Results are expressed as mean ± SEM of at least three separate experiments. Statistical analyses were done using Student’s t test to determine the significance of the differences between the various conditions.

### Software and tools used for ZFN design

- ZIFIT: It combines OPEN, CODA and modular assembly to screen for zinc finger proteins [23].
- SAPTA: Scoring algorithm for predicting TALEN activity. This tool evaluates and assigns scores of several target sites that give an estimate of predicted TALEN activities [24].
- TALEN-T: TAL effector nucleotide targeter v 2.0 (Cornell University). It is a tool for designing pairs of TALENs to target a specific gene sequence and screens for off-target effects [25].
- B1H screens of C2H2-ZF domains: predicting DNA-binding specificities for C2H2-ZF proteins [26].

## Results

### CompoZr ZFN pair targeting E6 gene demonstrated high editing activity in Yeast Mel I assay but not in cervical cancer cell lines

**Fig. 1:**
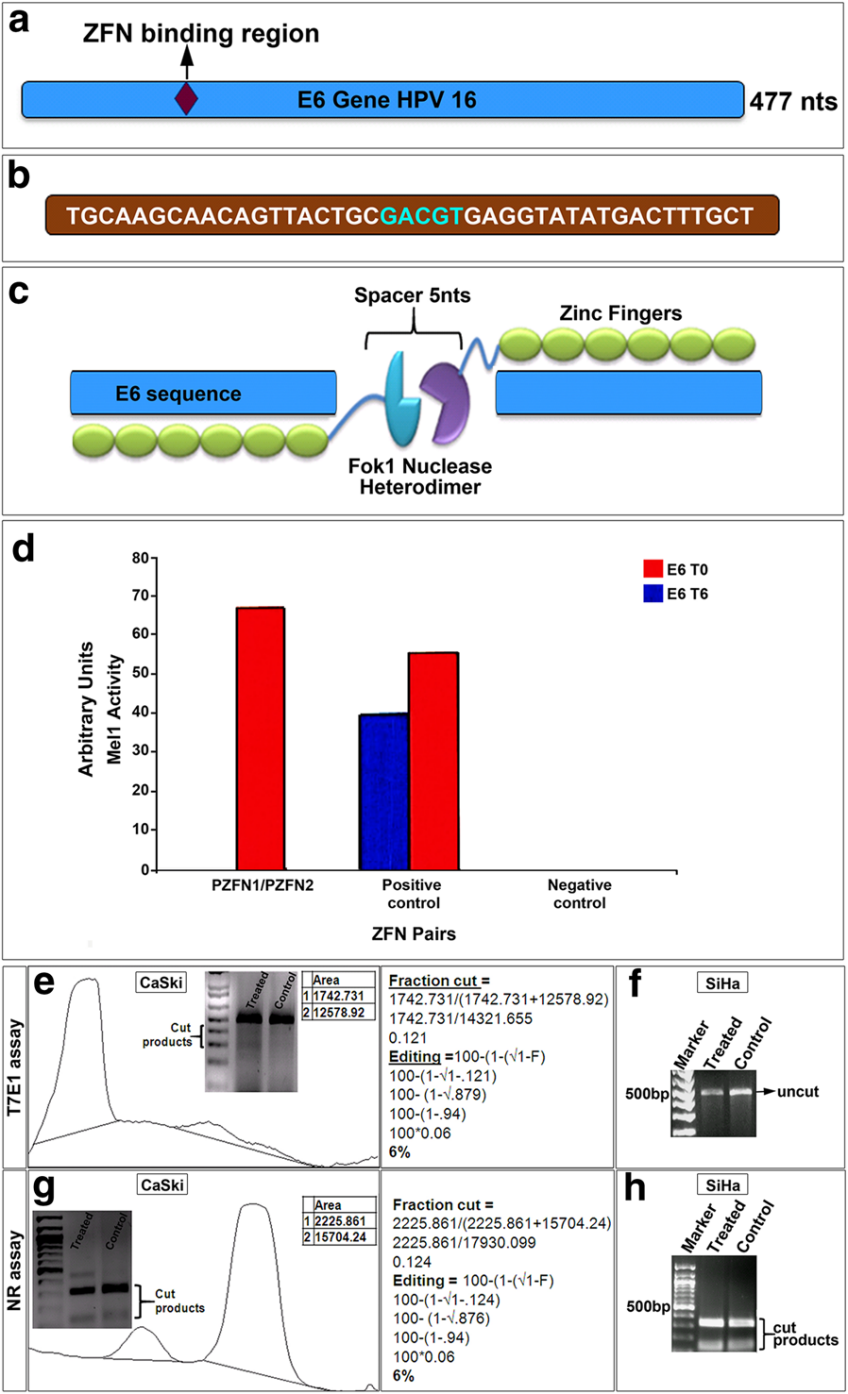
CompoZr ZFNs designed to target *E6* gene of HPV 16. **a** Schematic representation of ZFN targeting a region in exon 1 region of HPV 16-E6. **b** Represents the nucleotide sequence of the target region of E6. **c** An illustration of the designed ZFNs targeting *E6* region containing six ZFs on either side with a spacer region of 5 nucleotides. **d** Graph depicts yeast MEL-I assay showing activity of CompoZr ZFNs (red bar). T7E1 editing showing ~ 6% editing in CaSki cell line (**e**) and no editing in SiHa cell line (**f**) upon ZFN treatment. Further, complementary nuclease resistance (NR) assay also yielded ~ 6% editing in CaSki cell line (**g**), while NR assay did not yield any editing for SiHa cell line (**h**)

E6 gene is 477 bp long and it is known to have three exons and two introns [27]. For a simple gene disruption, the first criterion is to target coding exons towards the beginning of the gene which may create mutations leading to complete abolition of the gene and be less likely to generate truncated protein artifacts with residual biological activity. The second criterion is to screen for unique areas in the genome in order to minimize off-target effects [28]. Based on these design parameters, CompoZr ZFNs (Sigma Aldrich) were designed spanning a region of 117–159 nt in the *E6* gene sequence (Fig. 1a, b). This target site was checked for sequence similarity within human genome and showed no match to any genomic sequence. The nuclease pair targeting this region contained six fingers (ZFN amino acid sequence shown in Additional file 1: Figure S1A) on either side of the target site with a spacer region of five nucleotides (Fig. 1c) ZFN editing activity (arbitrary units in y-axis) was analyzed using Yeast based Mel-I assay which showed greater than 50% activity than that of the positive control at 6 h post-induction (Fig. 1d). With a high activity for Mel-I assay, we further assessed the gene editing activity of CompoZr ZFNs in HPV positive cell lines.

Before commencing our experiments on editing with HPV 16 E6 gene, we checked for the presence of HPV-16 in SiHa and CaSki cell lines. PCR analysis revealed the presence of both E6 and E7 genes in SiHa and CaSki cells which was absent in C33A, an HPV^−ve^ cell line (Additional file 1: Figure S1B). Presence of *E6* gene in SiHa and CaSki cell lines was further confirmed by PCR and sequencing. Sequencing of the portion of genomic DNA confirmed that indeed these cells contain HPV-*E6* and *E7* genes (Additional file 1: Figure S1C, D).

**Fig. 2:**
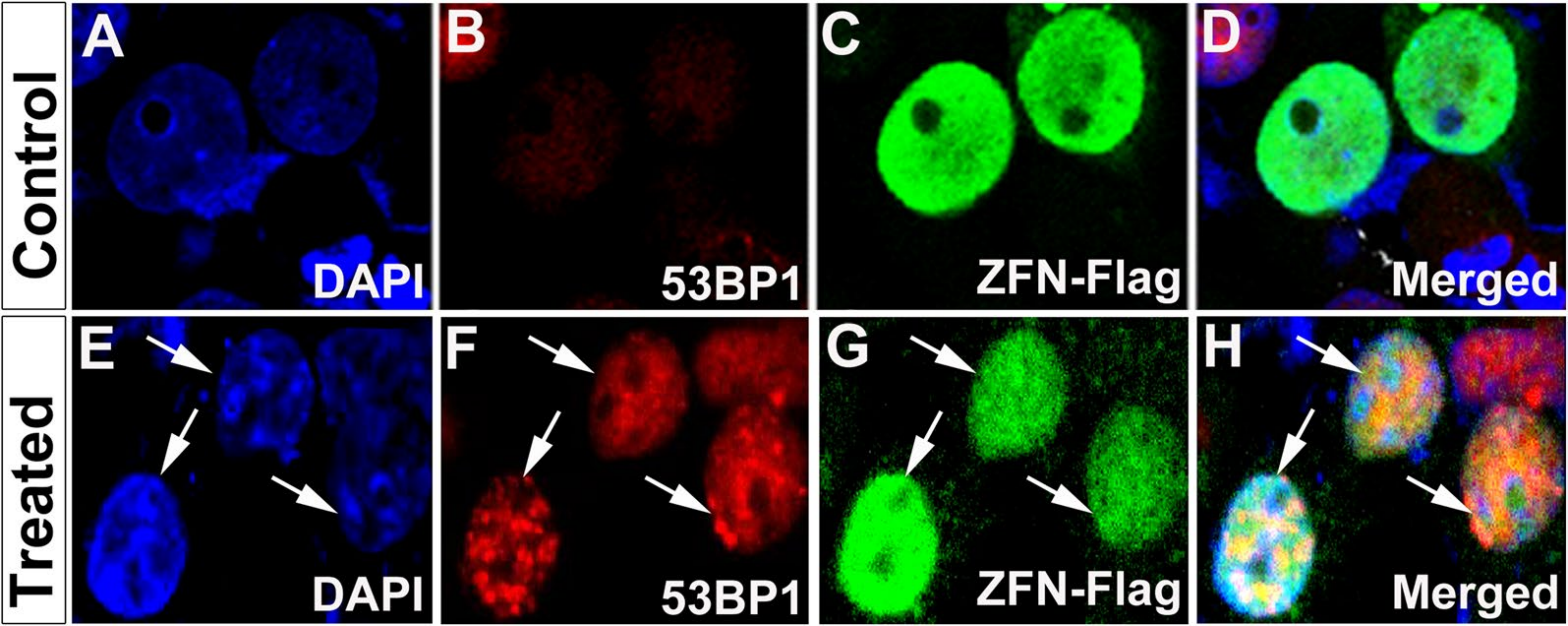
ZFN mediated editing indicated by 53bp1 (red spots) in CaSki cells. **A**–**D** Control cells with vector alone (green) showed no red spots (53bp1). **E**–**H** Treated cells with CompoZr ZFNs flag tagged (FITC-green) showed red spots indicating double strand breaks. Magnification ×600

**Fig. 3:**
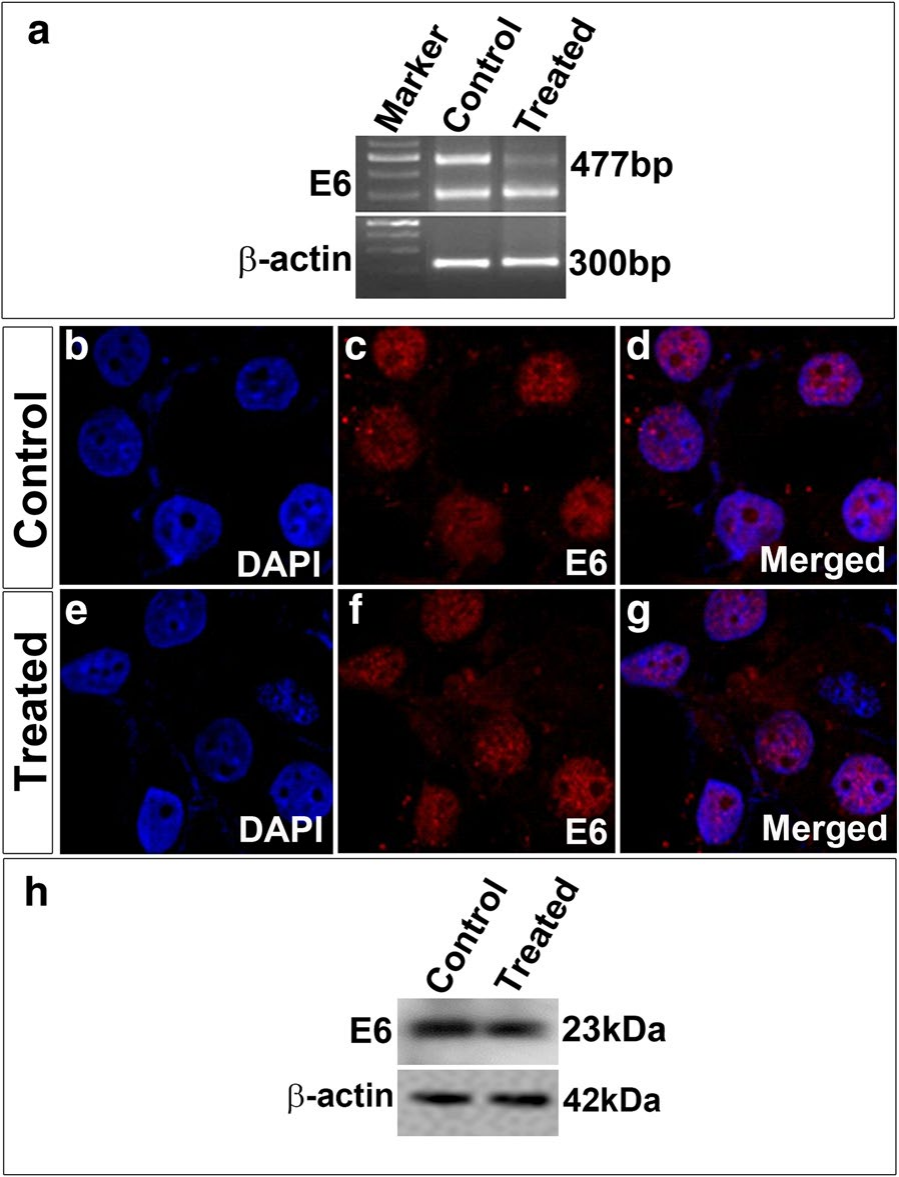
Expression analysis of E6 in CaSki cells showed no significant difference between control and treated groups. **a** RT-PCR data indicated that although full length E6 levels were decreased in treated, E6* spliced variant (300 bp) levels remained the same in both control and treated. This was further validated by immunocytochemical analysis for E6 proteins (**b**–**g**). ZFN treated CaSki cells showed no difference in E6 (red) levels after editing (**e**–**g**) when compared to control cells (**b**–**d**). Western blot analysis corroborated with the immunocytochemical analysis indicating no significant difference in E6 levels before and after treatment (**h**). Magnification ×600

Next, we transfected both SiHa and CaSki cells with ZFNs and obtained more than 70% efficiency in transfection in both the cells. Approximately 72 h post-transfection, cells were harvested and genomic DNA was extracted. Then a PCR was performed to amplify *E6* gene followed by T7E1 assay to detect NHEJ events. PCR products were amplified followed by denaturation and then gradual re-annealing of the products. T7E1 assay suggested that ~ 6% indels were obtained for *E6* in CaSki Cell line (Fig. 1e) whereas negligible activity (only ~ 0.1% indels) was obtained in SiHa cell line (Fig. 1f). These results were further corroborated by complementary nuclease resistance assay (NRA) which also showed a similar result for both CaSki (Fig. 1g) and SiHa cells (Fig. 1h). Further, immunocytochemical analysis for double strand breaks was done using 53bp1 antibody and very few cells containing ZFNs showed double stranded breaks in CaSki cell lines (Fig. 2A–H). We did not check in SiHa cells since these cells showed a negligible editing activity in T7E1 assay. With 6% editing in CaSki, we analyzed whether ZFN-mediated editing could actually reduce the *E6* levels in these cells. In order to check this, PCR for *E6* gene was carried out and did not find any editing (Fig. 3a). Further, immunocytochemical analysis was carried out with antibody specific to E6. We did not observe any significant difference in E6 levels in CaSki cell line after treatment when we checked by immunocytochemical (Fig. 3b–g) as well as western blot (Fig. 3h) analyses. This suggested that though editing could be observed at gene level, it was not reflected phenotypically.

We further treated cell lines with sodium butyrate to check if there is any interference due to methylation (Additional file 2: Figure S2A, B). Single stranded oligonucleotide (ssODN) treatment was done so that complementary nuclease assay could be done along with T7E1 assay to validate the efficacy of the treatments (Additional file 2: Figure S2C, D). Another group was treated with Nocodazole (Additional file 2: Figure S2E) synchronize all the cells to the same stage of cycle in order to enhance editing before sodium butyrate treatment. We observed that none these treatments improved the editing activity for E6 gene neither in SiHa nor in CaSki cells. Our data suggests that CompoZr ZFNs did not seem to be a successful strategy for E6 editing according to the RT-PCR results, but immunofluorescence assay indicated that some of the CaSki cells were observed to contain significant spots for 53BP1 which suggests that low editing efficiency masked the end point i.e. cells with no E6 phenotypic expression. Still, single cell cloning, and further analysis could probably yield a positive result.

**Fig. 4:**
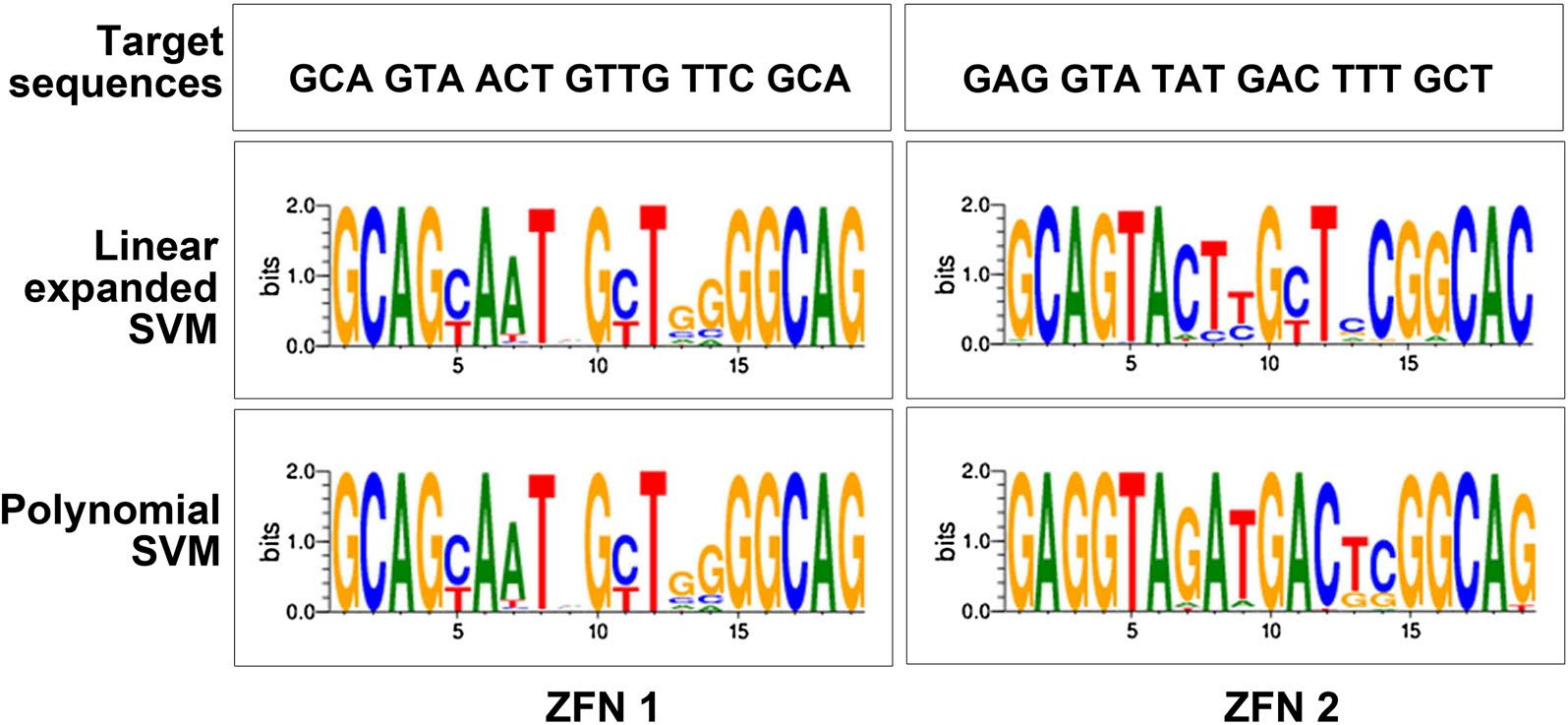
B1H screens of C2H2-ZF domains. B1H screens indicated that there was difference in the predicted binding site and the target sequence of CompoZr ZFN 1 and 2

Then we checked if CompoZr ZFN sequences bound to their target site. There have been many reports where ZFNs have failed to produce significant activity and there are many studies which study zinc finger protein binding to its target site. Recently, newer tools are emerging to assess and predict DNA binding to respective target sites. Hence, we performed an in silico analysis using B1-H screens which predicted that some of the modules in CompoZr ZFN 1 and 2 did not match exact binding to the target site (Fig. 4). This method takes into account additional DNA binding DNA–amino acid binding contacts, and higher order interactions that are responsible for specificity. Altogether, our data suggested that editing of HPV-E6 gene was not effective using CompoZr ZFNs.

### Screening for ZFNs targeting E6 of HPV 16 using ZIFIT and Zif Predict tools did not yield any active ZFN pairs

**Fig. 5:**
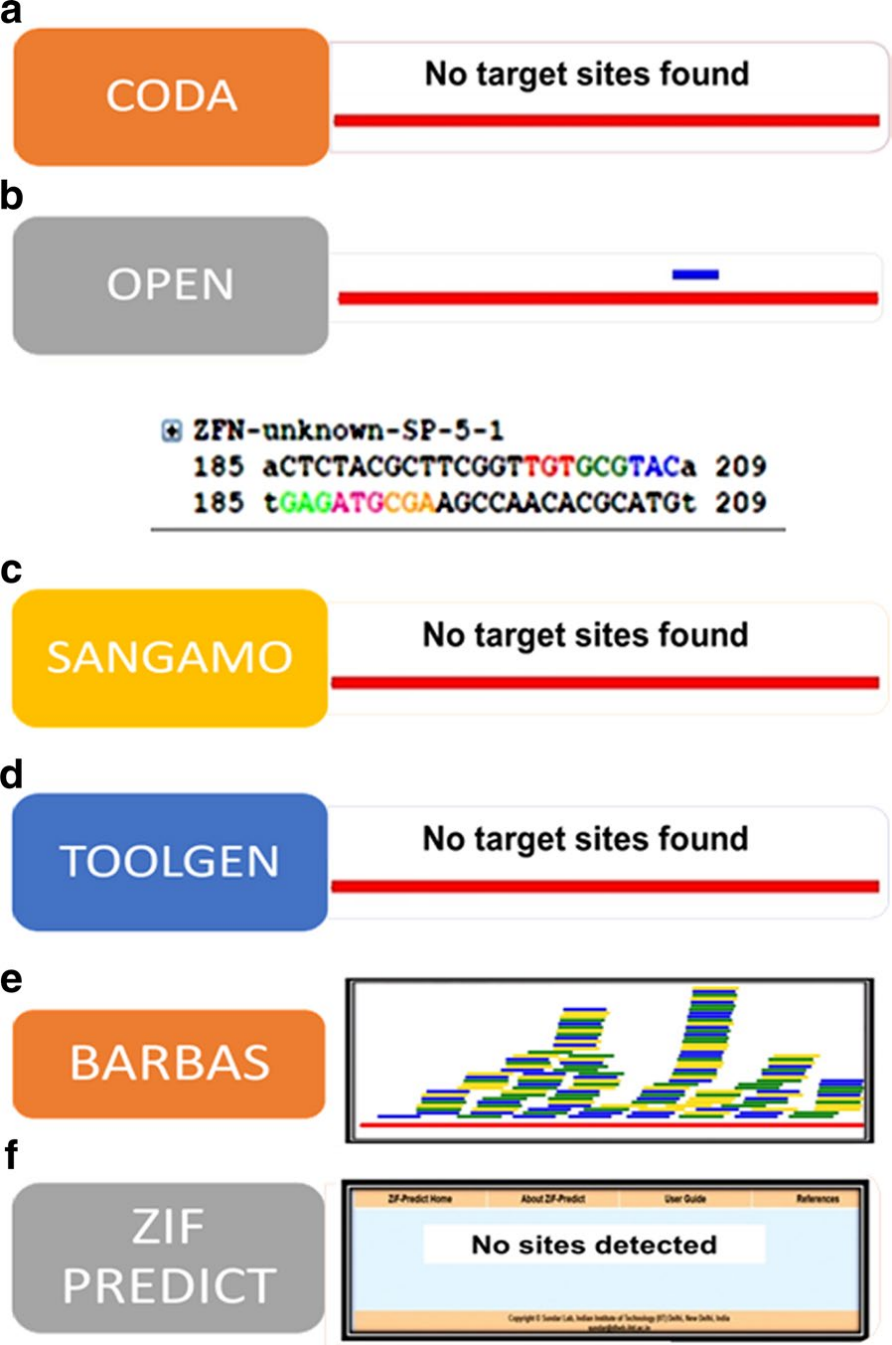
In silico ZFN target site prediction for *E6* gene using ZiFiT tools did not yield suitable target sites. E6 gene was screened for possible ZFN binding regions using **a** CoDa specific zinc fingers, **b** OPEN validated zinc fingers, **c** Sangamo validated modules, **d** TOOLGEN validated naturally occurring ZFN modules, **e** Barbas group of validated ZFN modules and **f** ZIF Predict

Since ZFNs designed to target E6 gene did not yield any activity, we then screened for other ZFNs to target *E6*. ZFNs targeting HPV-E6 were screened using various open access platforms such as CoDA, OPEN, Modular assembly, Sangamo, Toolgen, Barbas and Zif Predict (Fig. 5). CoDA and OPEN have been reported to give high success rates in editing. For E6 gene of HPV 16 CoDA (Context dependent assembly) that accounts for context dependency and utilizes well characterized N and C terminal finger pools, did not give any hits. OPEN, which has well characterized zinc finger arrays for GNN and TNN triplets [29] showed a few hits for E6 gene, but their prediction scores showed that they were inactive. Likewise, Modular assembly, Sangamo, Toolgen and Zif Predict also failed to yield any target sites for E6. For Modular assembly, it is well known that the likelihood for success depends on more number of GNN triplets as they have been very well validated. However, Barbas modules gave several ZFN target sites for E6 but only one of the half sites showed a high GNN score. All these analyses in our study indicated that identifying a proper ZFN pair to target HPV-E6 gene was difficult using publicly available tools. In silico analysis for ZFNs targeting E7 also did not yield any suitable hits (Additional file 3: Figure S3).

### TALEN pair designed to edit E7 gene showed editing in SiHa and CasKi cell lines

**Fig. 6:**
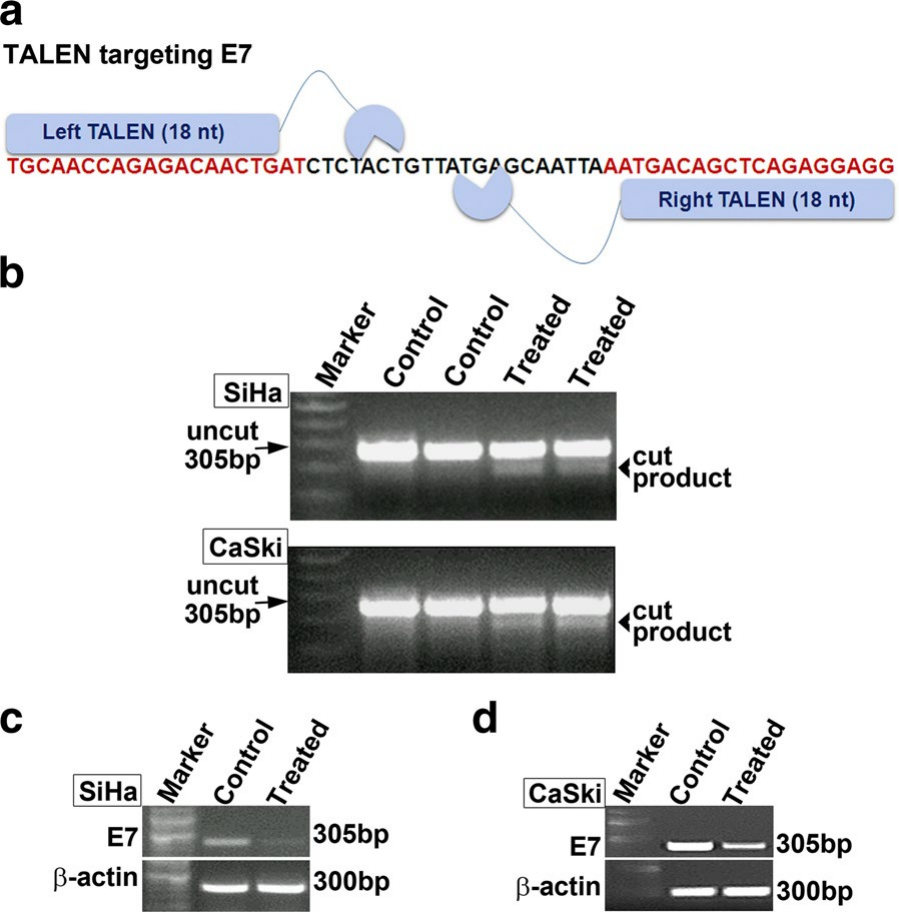
TALEN targeting E7 in SiHa and Caski cells showed reduction in *E7* levels. **a** Schematic representation of TALEN targeting E7. **b** T7E1 assay showing cut products in both SiHa and CaSki cell lines with TALEN treatment. Further, RT-PCR revealed a reduction in E7 levels in SiHa (**c**) and CaSki (**d**) cell lines

**Fig. 7:**
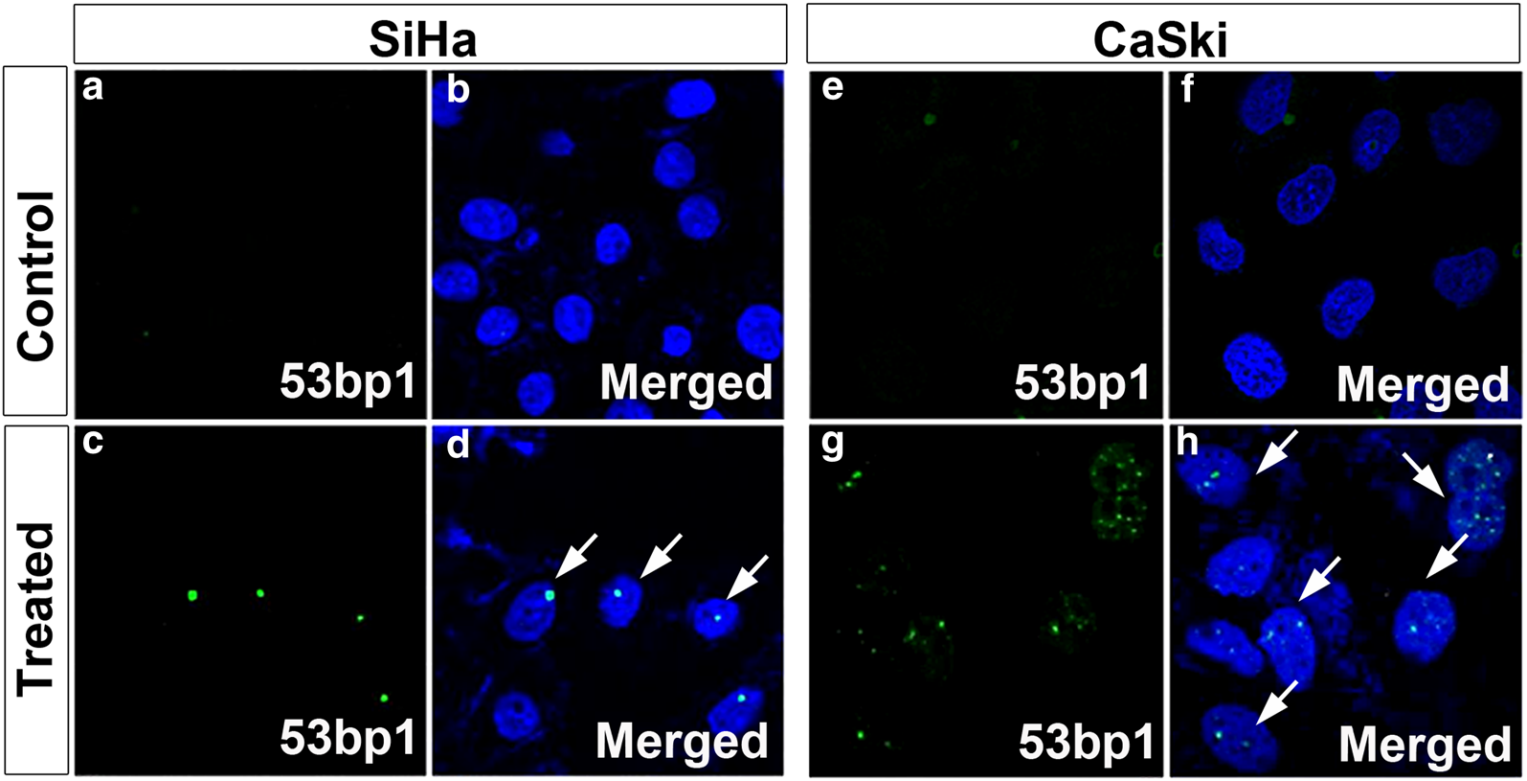
TALEN mediated editing in SiHa and CaSki cell lines. **a**, **b** SiHa control, **c**, d TALEN treated SiHa cells showed single spots (green; arrows) indicating the presence of 53bp1, **e**, **f** CaSki control, **g**, **h** TALEN treated CaSki cell showed multiple spots (green; arrows) indicating the presence of DNA double strand breaks. Magnification ×600

**Fig. 8:**
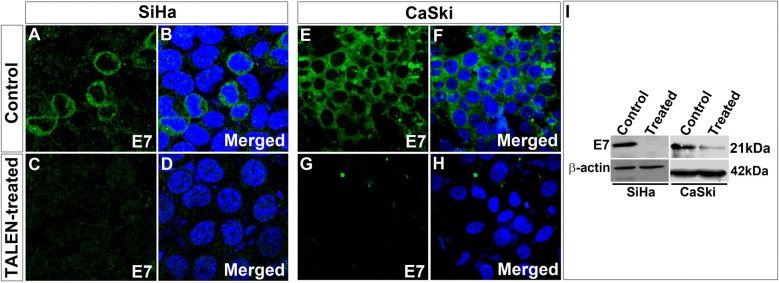
TALEN-mediated editing resulted in the reduction of E7 protein in both SiHa and CaSki cells. Immunocytochemical analysis revealed that levels of E7 protein was reduced in TALEN-treated (**C**, **D**) when compared to control (**A**, **B**) SiHa cells. Similarly, a reduction in E7 levels was observed in CaSki cells also after TALEN treatment (**G**, **H**) than in its respective control cells (**E**, **F**). This was further confirmed by Western blot analysis which showed a significant reduction in the levels of E7 after TALEN treatment in both the cell lines (**I**). Magnification-×600

We designed TALENs to edit *E6* gene. Gene editing efficiency of TALENs designed using SAPTA TALEN software targeting *E6* was assessed. In silico, Off-target effects for were checked using TALEN-T software and were found to be zero. However, T7E1 assay for TALENs targeting at position 112 did not yield any editing activity in both the cell lines (Additional file 4: Figure S4). We also designed TALENs targeting position 57 and 284 which also did not yield editing activity in T7E1 assay in both cell lines (Additional file 4: Figure S4). Hence, we designed TALENs to target *E7* gene. TALENs designed targeting the position 44 of *E7* (Fig. 6a) were also found to yield T7E1 editing activity in CaSki cells in vitro (Fig. 6b). We recently reported that TALENs successfully edit *E7* gene in SiHa cells [20], leading to necrotic cell death. This was further corroborated by RT-PCR analysis for the transcripts corresponding to *E7* in both SiHa and CaSki cells. In both the cells the expression of *E7* was drastically reduced in TALEN-edited group (Fig. 6c, d). Immunocytochemical analysis for double strand breaks using 53bp1 antibody further confirmed the efficient editing by the designed TALENs in both SiHa and CaSki cells (Fig. 7a–h). All these results pointed towards an efficient editing of E7 mediated by TALENs in both SiHa as well as CaSki cells. Subsequent immunocytochemical analysis for E7 proteins in both SiHa (Fig. 8A–D) and CaSki (Fig. 8E, F) cells revealed that the TALEN-mediated editing also resulted in the reduction of target E7 proteins in both the cell lines. This was further corroborated by western blot analysis which also showed a marked decrease in E7 proteins in both SiHa and CaSki cells (Fig. 8I). Thus, our results suggested that although TALENs were effective in editing in *E7* gene it was unable to target *E6* gene.

## Discussion

Gene editing using ZFNs and TALENs has been efficiently used in many systems. The ability to utilize a cell’s endogenous repair system has made it possible to make alternations in the gene in a site-specific manner. The duration and magnitude of nuclease expression is an important parameter for both on target and off-target nuclease activity. Recently, CRISPR/Cas system has dominated the gene editing field because it does not require engineering of proteins that bind to DNA in a specific manner. In our study TALEN targeting *E7* gene generated cut products for T7E1 assay and E7 levels were also reduced in both cell lines as indicated in the immunofluorescence and RT- PCR analyses. Our data revealed that TALEN-mediated editing of HPV-*E7* gene showed a reduction in *E7* levels after treatment in both SiHa and CaSki cell lines. However, treating cell lines with TALEN targeting *E6* did not give any editing activity for *E6* gene. It has also been showed by Sander et al. [30] that ZFNs failed to induce any mutations in the gene of interest. The ZFN and TALEN targeting E6 were designed at an exon–intron boundary. TALEN pair designed at exon–intron boundary did not yield any mutations in the target gene [31].

Several ZFN design platforms were screened for E6 gene using publicly available platforms. Since E6 gene had few GNN triplets it indicated that ZiFiT could not provide suitable hits. On similar lines, in a study by Wayengara, where zinc finger arrays were used to target HPV 16 and HPV 18 genome using ZiFiT software, only a set of unpaired zinc finger arrays of length 9 bp were obtained for *E6* gene [32]. It is known that zinc fingers that recognize *GNN* triplets are well characterized and have been used to successfully design ZFNs to knockout several genes. In silico analysis implied that limited GNN rich regions in *E6* and *E7* gene sequence of HPV 16 due to low GC content restricted the design options for ZFNs.

Since in silico tools did not yield suitable hits, we procured CompoZr ZFN for *E6* gene. Initial region of the *E6* gene was selected for gene knockout. CompoZr ZFN design for *E6* contained six fingers with more coverage of GNN triplets in the sequence. Genome editing includes many criteria such as number of fingers, spacer length and chromatin accessibility. Another critical factor to be considered is that the target exon should be present in all splice variants. Through this study, it was demonstrated by T7E1 and Nuclease resistance assay that CompoZr ZFNs showed some editing activity of *E6* gene in CaSki cells, but negligible activity in SiHa cell line. This could be attributed to the low editing levels of E6 gene exhibited by the ZFN pair. Further, there was no significant decrease in the levels of E6 in both the cell lines as shown by RT-PCR and immunofluorescence. Though the six-finger design had increased specificity, gene activity reduction due to additional fingers has been indicated in recent reports. It has been reported that the number of fingers and the number of nucleotides in the spacer region can affect the ZFN activity [33]. In their study, when they performed Cel I assay for 5 or 6 zinc fingers on either side showed no cleavage indicating that the ZFN pairs were not active. Similarly, when the spacer nucleotide size was 4, 5, 7 or 8, the activity of the 6 fingered ZFN pair was negligible. The ZFN pair used in our study had 6 fingers on each side with a spacer region of 5 nucleotides which could possibly be a reason for the low activity that we observed.

Although activity levels of ZFN in SiHa and CaSki were low, yeast Mel-I assay showed greater than 50% activity after 6 h of induction. A recent report by Yang et al. [34], showed that among the three pairs of ZFNs only one exhibited very significant high activity in yeast MEL-I assay, but apparently this pair failed to show any activity in their cell system. This suggests that ZFNs validated in yeast based assay may not necessarily be active in tumor-derived cell lines or in vivo. Even commercial designs do not often work as expected which can be observed from yeast Mel-I assay which showed reasonable editing activity, but showed low level of editing activity in cell lines.

Usually in two finger modules, binding of each module enhances cooperative and specific base recognition of subsequent modules. Considering this factor and further extrapolating our analysis on CompoZr ZFN modules indicate that binding affinity and specificity of modules for both ZFNs could affect improper binding to their target triplets. This in turn would affect Fok-I dimerization which in turn would lead to poor cleavage of target site (Additional file 5: Figure S5).

This study points to the fact that nucleases with low editing activity may be useful in making knockout cell lines that are derived using single cell cloning. However, studies which require mass reduction of a particular gene, editing activity should be good enough and also reflect phenotypically. Therefore, nucleases intended for therapeutic applications should necessarily have good editing activity so that downstream phenotypic effects are prominent. In addition to this, nuclease should also be optimized to reduce toxicity in such systems.

## Conclusions

Our study points to the fact that nucleases with low editing activity may be useful in making knockout cell lines that are derived using single cell cloning. However, studies which require mass reduction of a particular gene, editing activity should be good enough and also reflect phenotypically. Therefore, nucleases intended for therapeutic applications should necessarily have good editing activity so that downstream phenotypic effects are prominent. In addition to this, nuclease should also be optimized to reduce toxicity in such systems.

## Additional files

**Additional file 1: Figure S1.** Sequence analysis of HPV 16 *E6* and *E7* gene. (A) CompoZr ZFN sequences, (B) PCR showing the presence of *E6* and E7 in SiHa and CaSki cell lines. C33A, an HPV^−ve^ cell line was used as a negative control. Sequencing PCR for *E6* gene in SiHa (C) and CaSki (D) cell lines, respectively.

**Additional file 2: Figure S2.** Methylation status did not affect the editing efficiency of TALENs and ZFNs. SiHa and CaSki cells treated with 10 mM sodium butyrate showed some editing by both TALEN and ZFN in CaSki (A), but not in SiHa cell line (B). Treatment with ssODN and Sodium butyrate did not yield any editing in either of the cell lines (C, D). Nocodazole was used to bring cells to the same phase of cell cycle and then treated with ZFNs and TALENs along with ssODN. No significant editing was observed in editing after Nocodazole treatment in both the cells (E).

**Additional file 3: Figure S3.** In silico ZFN target site prediction for E7 using following tools did not yield suitable target sites. *E6* gene was screened for possible ZFN pairs using (A) CoDa specific zinc fingers, (B) OPEN validated zinc fingers, (C) Sangamo validated modules, (D) TOOLGEN validated naturally occurring ZFN modules, (E) Barbas group of validated ZFN modules and F) ZFN predict.

**Additional file 4: Figure S4.** TALEN designed against E6 could not yield editing in both SiHa and Caski cells. (A) Schematic of three TALEN binding sites on E6 gene. (B, C) TALEN targeting sequence at position 284 and its T7E1 assay in SiHa and CaSki cells showed no significant editing. (D, E) TALEN targeting sequence at position 57 and its T7E1 assay in SiHa and CaSki cells showed no significant editing. (F, G) TALEN targeting sequence at position 112 and its T7E1 assay in SiHa and CaSki cells showed no significant editing.

**Additional file 5: Figure S5.** Improper binding of the designed CompoZr ZFNs led to poor editing of E6 in SiHa and CaSki cell lines. Binding of ZF modules is co-operative binding and since both the CompoZr ZFNs have some of the modules not binding to its predicted target site. This could have probably led to poor cleavage as was observed in the low cleavage efficiency obtained in SiHa and CaSki cell lines.
