# Supplementary figures and images for: Genome editing of oncogenes with ZFNs and TALENs: caveats in nuclease design
Source: Cancer Cell Int. 2018 Oct 22;18:169. doi: 10.1186/s12935-018-0666-0 (PMC6198504; doi:10.1186/s12935-018-0666-0)

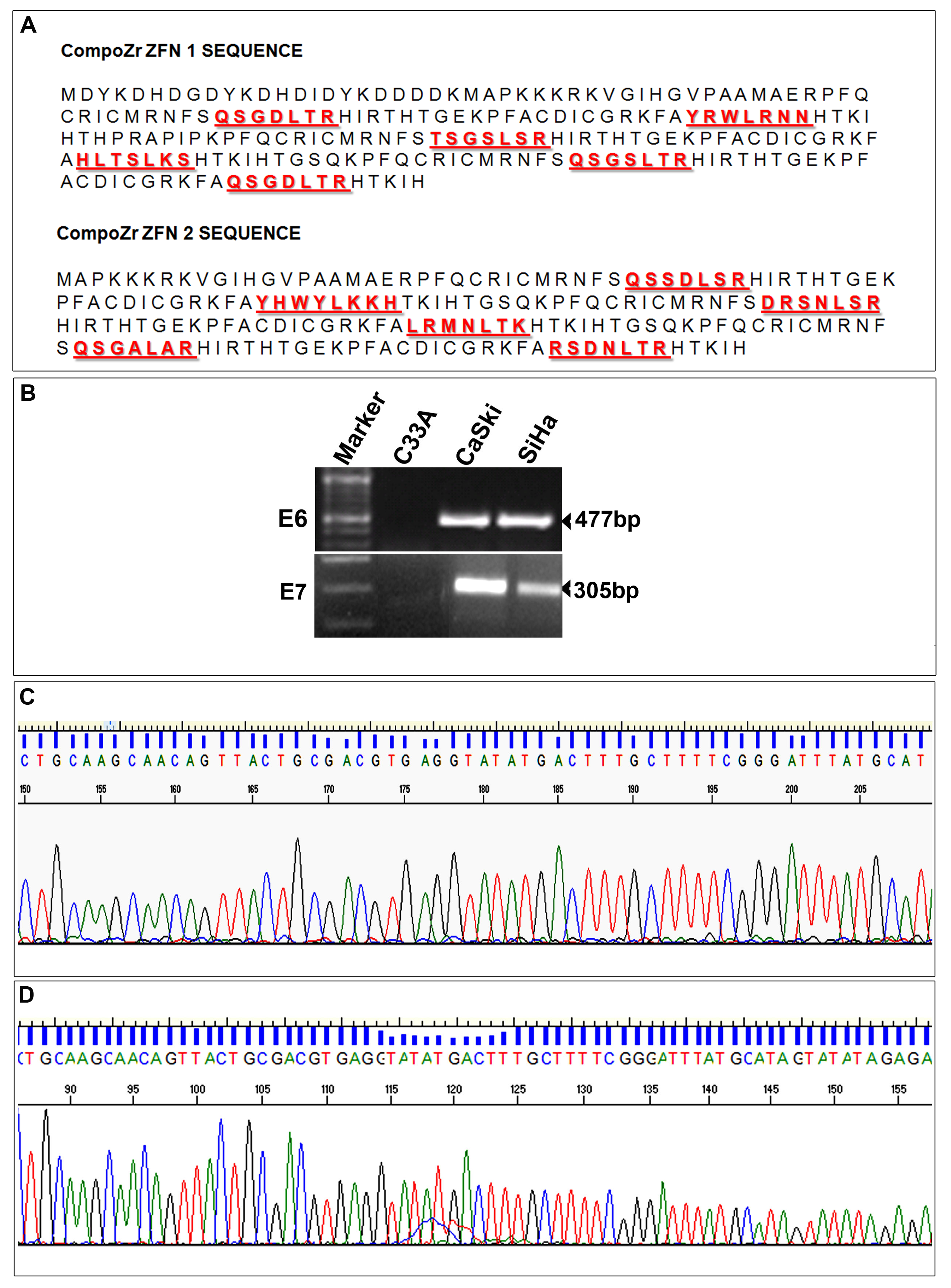

Supplement: Supplementary file 1 — Additional file 1: Figure S1. Sequence analysis of HPV 16 E6 and E7 gene. (A) CompoZr ZFN sequences, (B) PCR showing the presence of E6 and E7 in SiHa and CaSki cell lines. C33A, an HPV−ve cell line was used as a negative control. Sequencing PCR for E6 gene in SiHa (C) and CaSki (D) cell lines, respectively. [file 12935_2018_666_MOESM1_ESM.tif]

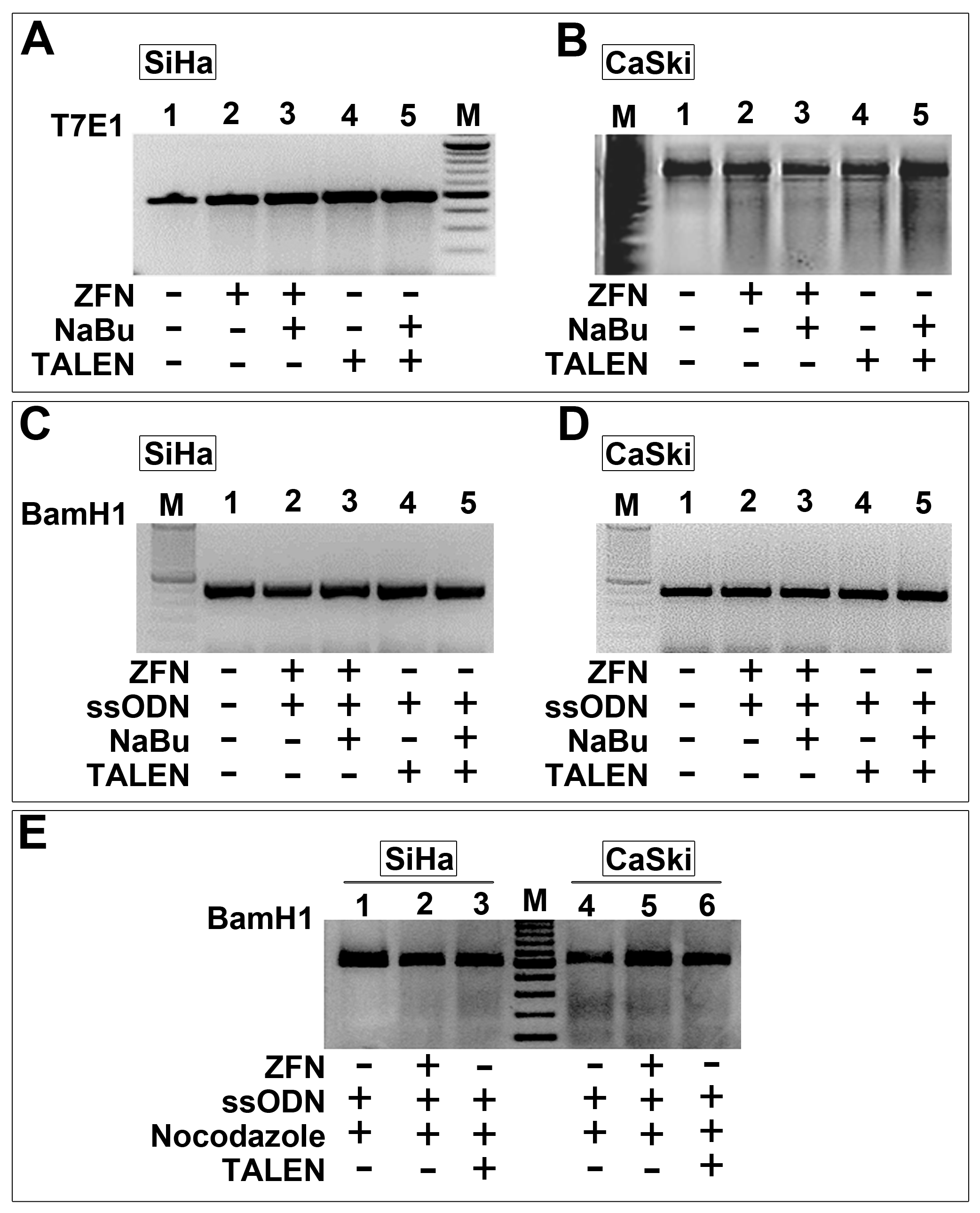

Supplement: Supplementary file 2 — Additional file 2: Figure S2. Methylation status did not affect the editing efficiency of TALENs and ZFNs. SiHa and CaSki cells treated with 10 mM sodium butyrate showed some editing by both TALEN and ZFN in CaSki (A), but not in SiHa cell line (B). Treatment with ssODN and Sodium butyrate did not yield any editing in either of the cell lines (C, D). Nocodazole was used to bring cells to the same phase of cell cycle and then treated with ZFNs and TALENs along with ssODN. No significant editing was observed in editing after Nocodazole treatment in both the cells (E). [file 12935_2018_666_MOESM2_ESM.tif]

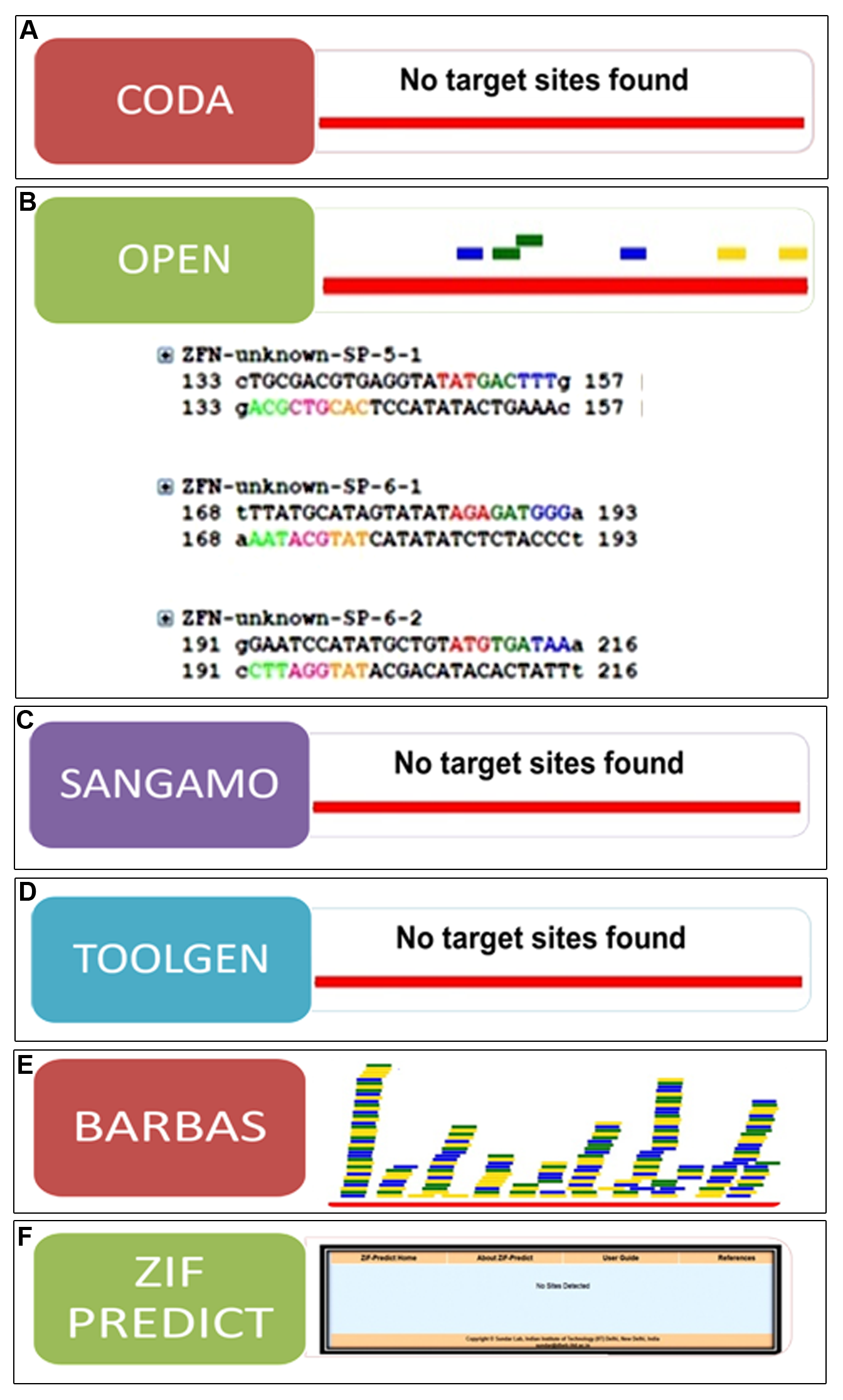

Supplement: Supplementary file 3 — Additional file 3: Figure S3. In silico ZFN target site prediction for E7 using following tools did not yield suitable target sites. E6 gene was screened for possible ZFN pairs using (A) CoDa specific zinc fingers, (B) OPEN validated zinc fingers, (C) Sangamo validated modules, (D) TOOLGEN validated naturally occurring ZFN modules, (E) Barbas group of validated ZFN modules and F) ZFN predict. [file 12935_2018_666_MOESM3_ESM.tif]

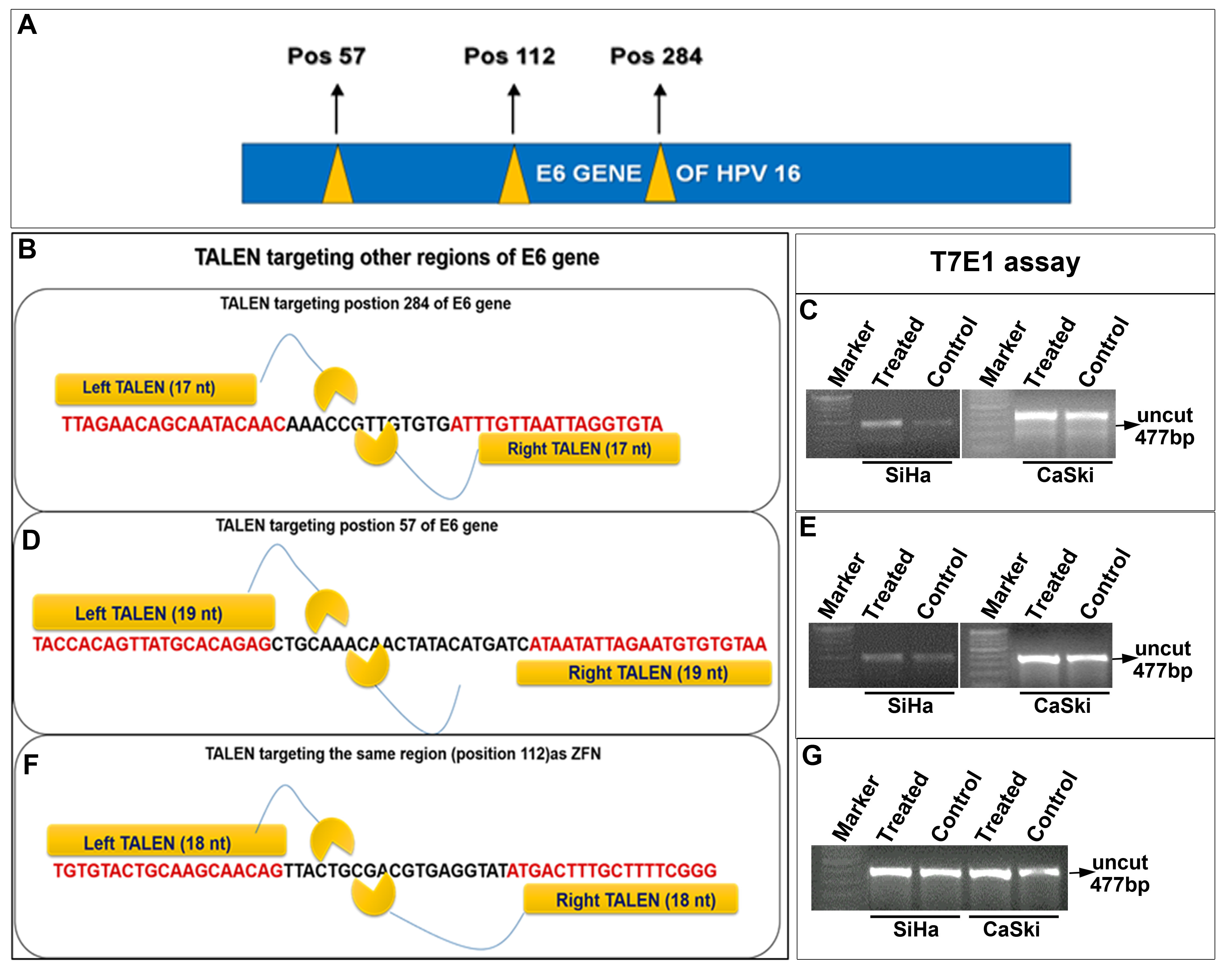

Supplement: Supplementary file 4 — Additional file 4: Figure S4. TALEN designed against E6 could not yield editing in both SiHa and Caski cells. (A) Schematic of three TALEN binding sites on E6 gene. (B, C) TALEN targeting sequence at position 284 and its T7E1 assay in SiHa and CaSki cells showed no significant editing. (D, E) TALEN targeting sequence at position 57 and its T7E1 assay in SiHa and CaSki cells showed no significant editing. (F, G) TALEN targeting sequence at position 112 and its T7E1 assay in SiHa and CaSki cells showed no significant editing. [file 12935_2018_666_MOESM4_ESM.tif]

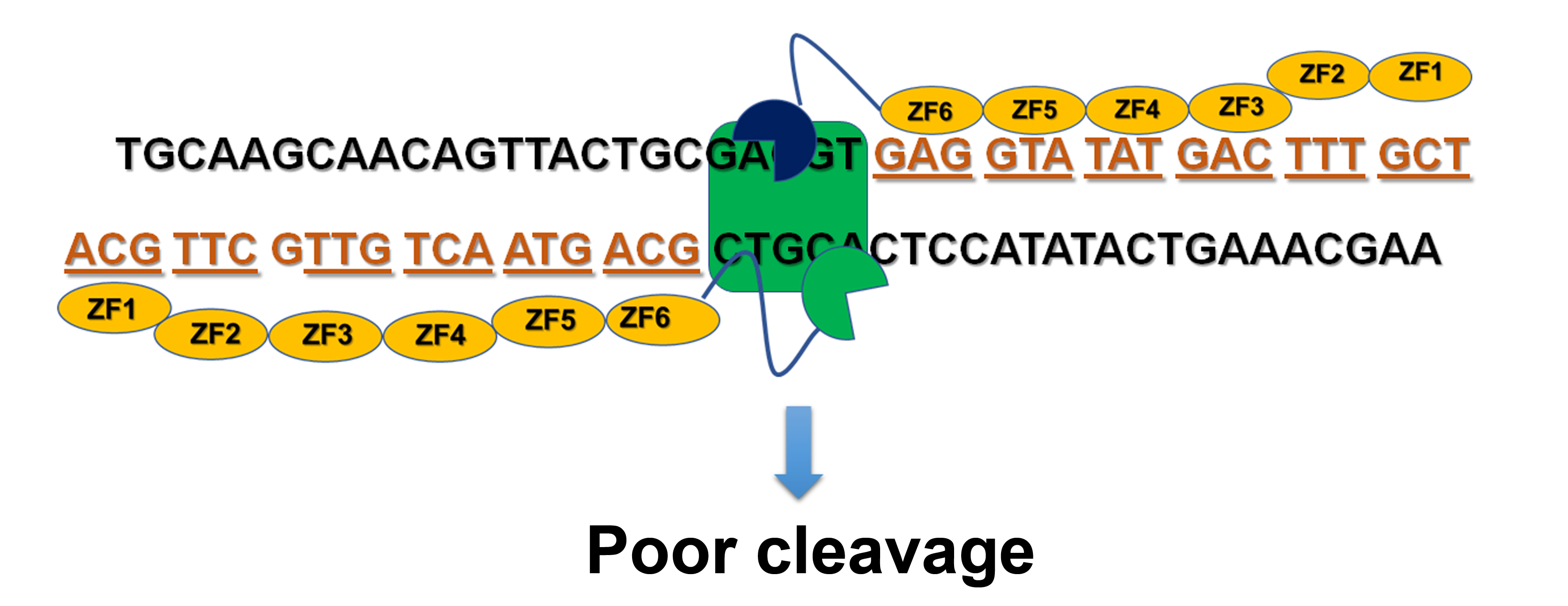

Supplement: Supplementary file 5 — Additional file 5: Figure S5. Improper binding of the designed CompoZr ZFNs led to poor editing of E6 in SiHa and CaSki cell lines. Binding of ZF modules is co-operative binding and since both the CompoZr ZFNs have some of the modules not binding to its predicted target site. This could have probably led to poor cleavage as was observed in the low cleavage efficiency obtained in SiHa and CaSki cell lines. [file 12935_2018_666_MOESM5_ESM.tif]
